# Identification of Functional and Druggable Sites in *Aspergillus fumigatus* Essential Phosphatases by Virtual Screening

**DOI:** 10.3390/ijms20184636

**Published:** 2019-09-19

**Authors:** Benjamin P. Thornton, Anna Johns, Reem Al-Shidhani, Sandra Álvarez-Carretero, Isabelle S. R. Storer, Michael J. Bromley, Lydia Tabernero

**Affiliations:** School of Biological Sciences, Faculty of Biology, Medicine and Health, University of Manchester, Manchester Academic Health Science Centre, Manchester M13 9PT, UK

**Keywords:** protein phosphatases (PPases), phosphatase inhibitors, antifungals, *Aspergillus fumigatus*, computational screening, virtual screening (VS), VSpipe, ligand efficiency indices (LEIs), drug discovery

## Abstract

Fungal diseases are a serious health burden worldwide with drug resistance compromising efficacy of the limited arsenal of antifungals available. New drugs with novel mechanisms of action are desperately needed to overcome current challenges. The screening of the *Aspergillus fumigatus* genome identified 35 phosphatases, four of which were previously reported as essential for viability. In addition, we validated another three essential phosphatases. Phosphatases control critical events in fungi from cell wall integrity to cell cycle, thus they are attractive targets for drug development. We used VSpipe v1.0, a virtual screening pipeline, to evaluate the druggability of the seven essential phosphatases and identify starting points for drug discovery. Targeted virtual screening and evaluation of the ligand efficiency plots created by VSpipe, enabled us to define the most favourable chemical space for drug development and suggested different modes of inhibition for each phosphatase. Interestingly, the identified ligand binding sites match with functional sites (active site and protein interaction sites) reported for other yeast and human homologues. Thus, the VSpipe virtual screening approach identified both druggable and functional sites in these essential phosphatases for further experimental validation and antifungal drug development.

## 1. Introduction

Fungal diseases are an ever-increasing burden to health services worldwide with approximately 1.2 billion people suffering from some form of fungal infection [1]. The majority of these cases are the result of superficial, albeit problematic, fungal infections of the skin or mucosa, yet between 1.5 and 2 million deaths are caused each year by systemic infections [2]. Only four classes of drugs are currently recommended for the treatment of invasive diseases, with the azole class recommended for primary therapeutic purposes in many instances. Resistance to azoles is emerging rapidly in some key pathogens, particularly *Aspergillus fumigatus*. For individuals infected with a resistant isolate of *A. fumigatus*, the mortality rate increases from ca. 50% to almost 90%. New targets and therapeutic classes are urgently needed to overcome the current challenges in treating fungal diseases.

Fungal protein phosphatases are involved in critical cellular functions and regulate cell wall integrity, metabolism, calcium homeostasis, and cell cycle control amongst others [3,4,5]. Phosphatases such as Aspergilus protein phosphatase Z PpzA, calcineurin, serine/threonine phosphatase Sit4, dual-specificity phosphatase YVH1, and PtcB, a high osmolarity glycerol response (HOG) phosphatase, also play important roles in virulence and drug resistance in *A. fumigatus* and *Candida albicans* infections [6,7,8,9,10]. Furthermore, inhibitors of calcineurin have shown synergism with current antifungals against invasive fungal infections [7,11,12]. Therefore, these enzymes are emerging as promising targets for the development of new classes of antifungals that could alleviate the current bottleneck in drug development in this space.

Recently, a genome-wide evaluation of protein phosphatases in *A. fumigatus* highlighted at least four enzymes that are required for viability of this organism and the role of some of them in high osmolarity regulation and iron metabolism [13]. However, these phosphatases remain largely uncharacterised and structural information about *A. fumigatus* phosphatases (AfPPases) is still lacking, which limits the full exploitation of these targets for drug discovery.

In this study, we used a combination of computational and experimental approaches to expand the ontology-based classification and previous identification of AfPPases [10,14], and we validated three additional phosphatases as essential for viability. Finally, we used a virtual screening pipeline, VSpipe v1.0 [15] to define putative functional and druggable sites in the essential phosphatases. Results from the virtual screening suggest that different modes of inhibition may be possible to block phosphatase function and guide the selection of suitable starting points for drug development.

## 2. Results

### 2.1. Identification and Classification of Fungal Phosphatases

A total of 32 protein phosphatases have previously been identified in the genome of the human pathogenic fungus *A. fumigatus* using an early version of a phosphatase ontology based on domain architecture scanning [14], and by bioinformatics analyses [10]. In this study, we performed a de novo evaluation of the *A. fumigatus* (AF293) genome using an improved phosphatase ontology classification tool previously used to describe the TriTryp phosphatome [16]. Our analysis identified three new protein phosphatases, described here for the first time, as well as confirmed the 32 proteins already described (Supplementary Table S1). Two of the new phosphatases belong to the protein tyrosine phosphatase family, a phosphatase and tensin homologue PTEN-like (AfuA_2g11990, TepA) and a myotubularin-like phosphatase (AfuA_1g05640, YmrA). The third protein belongs to the serine threonine phosphoprotein phosphatase (PPP) family (AfuA_5g08620, PpefA).

In total we identified 35 phosphatases: 20 serine/threonine phosphatases (STPs) and 15 protein tyrosine phosphatases (PTPs), including seven putative dual-specificity phosphatases (DUSPs) and three putative lipid phosphatases. Putative orthologues of the *A. fumigatus* protein phosphatases were also identified in two other dominant fungal pathogens *C. albicans* and *Cryptococcus neoformans* (see Supplementary Table S1).

### 2.2. Characterisation of the Essential Phosphatase Cohort in A. fumigatus

Proteins that are essential for viability represent potential targets for the development of novel antifungal drugs. Previously, four protein phosphatase encoding genes (*glcA*, *pphB*, *fcpA*, and *dspC*) had been described as essential for cell viability [13]. Three other phosphatases (*ppgA*, *ssuA,* and *nimT*) have been proposed to be required for viability of *A. fumigatus*, although this has not been confirmed experimentally [13].

**Figure 1:**
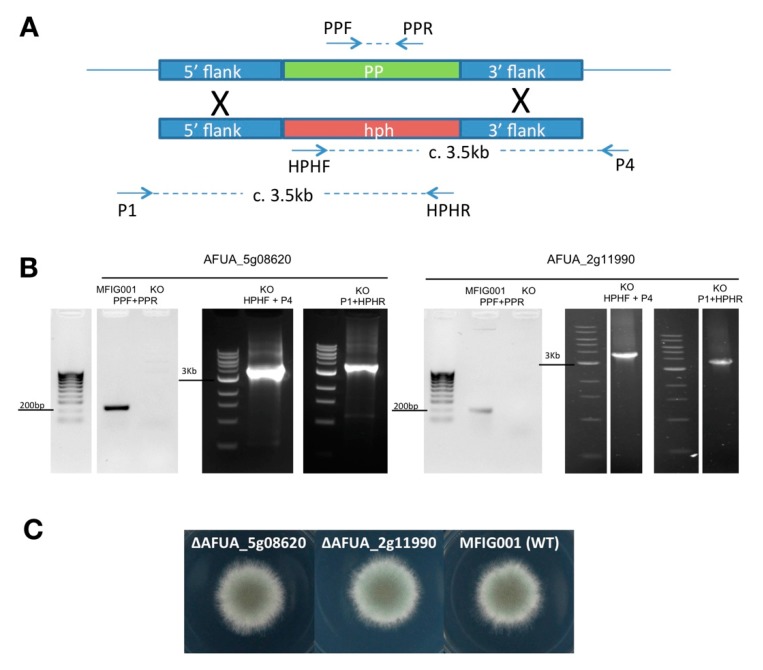
Protein phosphatase gene disruption. (**A**) Schematic representation of protein phosphatase (PP) gene replacement with a hygromycin-selectable marker (hph). The position of oligonucleotide primers used for validation in panel B, are given as arrows with indicative sizes of PCR amplicons shown between (sequences are listed in Supplementary Table S2). (**B**) PCR validation of the AFUA_5g08620 and AFUA_2g11990 null mutants. (**C**) Phenotype of the isogenic wild-type MFIG001 alongside the AFUA_5g08620 and AFUA_2g11990 null mutants on *Aspergillus* minimal media (48 h).

To assess whether any of the new phosphatase genes identified in this study (*ppefA*, *ymrA*, and *tepA*) are required for viability in vitro, we attempted to generate null mutants for all three genes. For two of them (*ppefA* and *tepA*), we were able to identify null homokaryotic isolates, which suggests that these protein phosphatases are dispensable for viability in vitro (Figure 1). However, we were only able to obtain balanced heterokaryons isolates for the third gene, AFUA_1g05640 (*ymrA*), suggesting it may be important for viability.

We then assessed whether *ppgA*, *ssuA*, and *nimT* are required for viability in *A. fumigatus*; for this, we attempted to perform gene replacement in a similar way as above. For one of these genes, *ppgA* (AFUA_5g11370), we were able to identify a viable, albeit poorly growing homokaryotic null mutant. For *ssuA* (AFUA_2g03760) and *nimT* (AFUA_6g08200), we were only able to identify heterokaryons carrying the selective marker, indicating they are essential for viability. Overall, we identified seven *A. fumigatus* phosphatase genes as important for viability and, therefore, potential new targets for drug development.

### 2.3. Building Molecular Models for A. fumigatus Phosphatases

Our approach to assess the druggability of the AfPPases was based on a combination of virtual screening with VSpipe [15] and pocket analysis with PockDrug [17] in order to identify suitable druggable sites for drug development on the essential AfPPases. Currently, there are no available 3D structures of any of these AfPPases, thus, we had to generate molecular homology models for the virtual screening. We first identified the closest homologue with a 3D structure available, using the sequence of each AfPPAse in a Blast search [18] (https://blast.ncbi.nlm.nih.gov/Blast) with the option to identify relatives with structures in the Protein Data Bank (PDB) ([19] https://www.rcsb.org/). For each of the target phosphatases, we identified at least one homologue with a structure available (Table 1). We then established the boundaries of the phosphatase catalytic domains using the sequence alignment with the template structures (Supplementary Figure S1). These were evaluated using secondary structure and disorder predictions and edited when needed to eliminate predicted disordered regions at the N- or C-terminal ends (see Section 4 for details).

**Figure 2:**
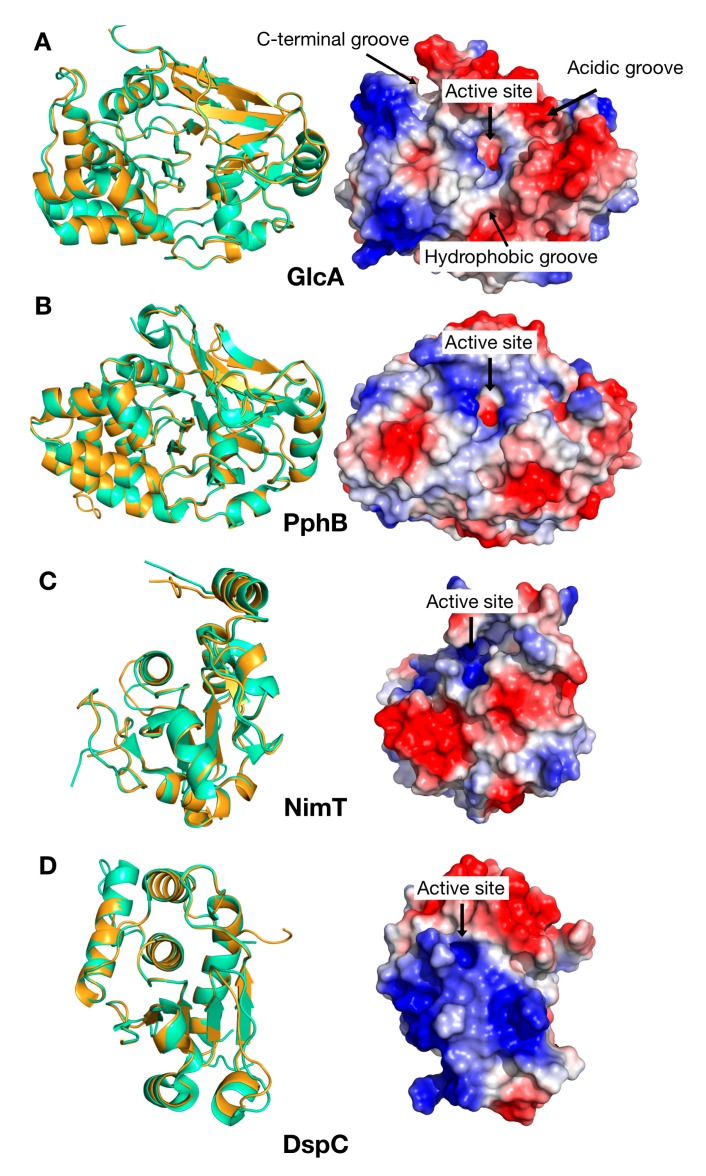
Homology models for the catalytic domains of the AfPPases. (**A**) GlcA model. (**B**) PphB model. (**C**) NimT model. (**D**) DspC model. Ribbon diagrams are shown on the left—orange for the AfPPases, green for the template. Electrostatic surface representations are shown on the right. Active sites are labelled for all phosphatase catalytic domains. Significant sites defined for the human protein phosphatase 1 (hPP1) are also labelled in (**A**).

For GlcA, a number of structures are available for various protein phosphatase 1 (PP1) isoforms (α, β, γ), with the human catalytic domain of protein phosphatase 1 β orthologue (PPP1CB) being the closest orthologue. A homology model for GlcA was then created using the advance option in Modeller [20] and the PDB structures 4G9J, 5IOH, and 1S70 as templates (Table 1). Similarly to the human PP1, the GlcA model surface shows three distinct grooves: The Acidic groove, the C-terminal groove, and the Hydrophobic groove at the bottom of the active site (Figure 2). For PphB, the closest homologue is the human catalytic domain of protein phosphatase 2 β isoform (PPP2CB) with 84% identity, but only the crystal structure of the hPP2A α isoform is currently known. The human PP2Aα structures, 2NYl, 3DW8, and 2NYM, were used as templates to generate the model with the advanced Modeller option as above. For NimT, the closest homologue is the human cell division control protein 25 (CDC25), for which structures of three isoforms (a, b, and c) are available (1C25, 1QB0, and 3OP3), thus all of them were used to generate the homology model. For DspC, the closest homologue structure available is that of Yvh1 from *Chaetomium thermophilum* (5M43), which was used as a template.

Building models for SsuA, YmrA, and FcpA was problematic, as these phosphatases contain long insertions (20 residues or more) at various regions in the *Af* sequence, not present in the template structures (Table 1), resulting in unstructured disordered loops in the final models (Supplementary Figure S2). We decided not to use these three models (SsuA, YmrA, and FcpA) for targeted virtual screening because the undetermined regions could bias the ligand docking (i.e., create artificial binding sites or block them). For example, the results of the blind docking with the SsuA (Aspergillus SSU72 homologue) model showed that most of the ligand clusters bind to sites near the disordered loops (Supplementary Figure S2).

### 2.4. Identification of Ligand Binding Sites by Virtual Screening

We have previously demonstrated that the virtual screening tool VSpipe could be used to identify functional ligand binding sites in the human phosphatase PTP1B, as well as to guide the selection of initial hits for drug discovery [15]. The *blind docking* option in VSpipe is useful when there is no previous knowledge about the functional sites on the target protein, as is the case for the AfPPases. The models of the two STPs: GlcA, PphB, and two DUSPs: NimT, DspC were screened using blind docking with a 500-fragment library (Maybridge_Ro3_Fragment_Library).

**Figure 3:**
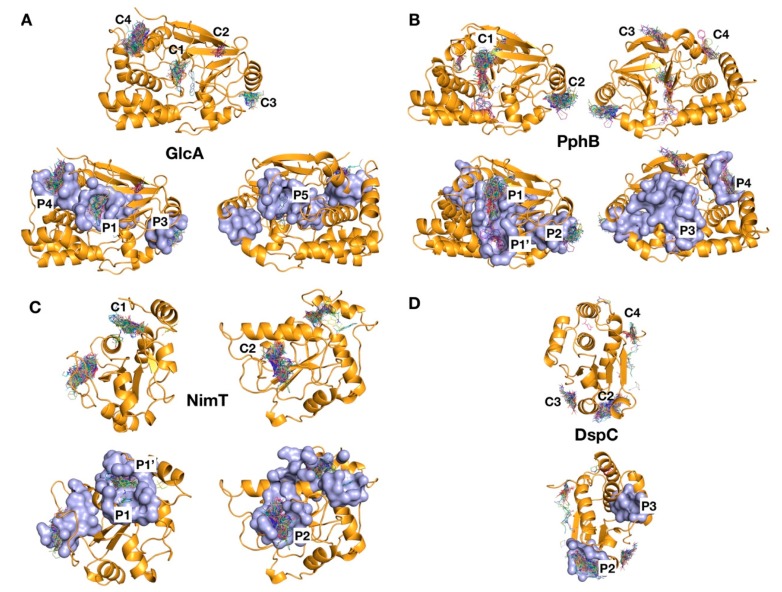
Virtual screening (VS) ligand clusters and druggable pockets for the AfPPases. Molecular models are shown in orange cartoons, ligands as lines, and druggable pockets in blue surface rendering. (**A**) VS results for GlcA, top: ligand clusters from VSpipe blind docking; bottom: ligand clusters and druggable pockets superimposed on the ribbon diagram; bottom left: front view, bottom right: back view. (**B**) VS results for PphB, top ligand clusters from blind docking, top left: front view, top right: back view; bottom: ligand clusters and druggable pockets superimposed on the ribbon diagram; bottom left: front view, bottom right: back view. (**C**) VS results for NimT, top: ligand clusters from VSpipe blind docking; top left: front view, top right: side view; bottom ligand clusters and druggable pockets superimposed on the ribbon diagram; bottom left: front view, bottom right: side view. (**D**) VS results for DspC, top ligand clusters from VSpipe blind docking; bottom ligand clusters and druggable pockets superimposed on the ribbon diagram.

The results were then used to identify initial ligand binding sites on the protein surface. Several compound clusters (clusters defined as 10 or more fragments in the same binding site) were found for each of the AfPPases (Figure 3). Four clusters were identified for GlcA and marked as C1, C2, C3, and C4. Three of those clusters map with functional sites described for the structure of hPP1—C1 at the active site, C2 in the acidic groove, and C4 at the C-terminal groove (Figure 3). C3 binds to a yet uncharacterised site on the side of the protein. Four clusters were also identified for PphB (Figure 3). Cluster C1 binds to the active site, C2 binds to a similar site in GlcA (C3), and C3 and C4 bind at the back of the protein.

Two clusters were identified for NimT: C1 at the active site and C2 away from the active site. The C2 site matches with a previously identified kinase-binding site in CDC25B [21]. The blind docking of DspC resulted in the identification of three main clusters, C2 and C3 at the bottom of the protein respect to the active site, and C4 on the side (Figure 3). No clusters were found at the active site, which in the template structure has a very narrow opening. The functional relevance of the binding sites identified is unknown, as no information is available for the related fungal or human DUSPs.

In sum, for each of the AfPPases, except for DspC, the virtual screening with a small library of compounds identified ligand-binding sites that match with reported functional sites (active site or protein–protein interaction) in the homologous proteins, suggesting that these may also have functional relevance in the uncharacterised AfPPases.

### 2.5. Surface Pocket Prediction

Next, we used the PockDrug-Server [17] to predict druggable pockets on the surface of each AfPPase. Several surface pockets were predicted, but only those pockets with druggability scores > 0.5 were considered for analysis (Table 2). In GlcA, three of the four predicted ligand clusters match druggable pockets (Figure 3A). These are the active site (P1), a side pocket (P3), and the C-terminal groove (P4) (Table 2). P5, at the back of the protein, matches with a low-density cluster (<10 compounds). For PphB, druggable pocket predictions (P1 and P2) agree with two clusters, active site C1 and side cluster C2 (Table 2 and Figure 3B), P3 at the back of the protein has no match to ligand clusters and P4, matches C4 (Figure 3B).

For NimT, pockets P1/P1′ and P2 match the C1 and C2 ligand clusters, respectively. Where P1 is the active site and extends beyond to a secondary pocket (P1′) above the active site (Figure 3C). The P2 pocket maps into the reported protein–protein interaction site in CDC25B [21]. For DspC, we identified two druggable pockets (Table 2), where only one of them, P2, matches a ligand cluster (C2) located at the bottom of the protein (Figure 3D). The second pocket, P3, is at the back, respective to the active site. The functional relevance of these pockets is unclear. We found no pocket at the active site in agreement with the results from the blind docking.

Similarly to the ligand clusters, the druggable pockets also map to functionally relevant regions in the human PPase orthologues, including the active site (P1) and other protein–protein interaction sites (discussed later). The matching of the virtual screening (VS) clusters and druggable pockets underscores the importance of the ligand binding sites identified and their potential for drug development.

### 2.6. Analysis of Targeted Docking at Druggable Sites

The blind docking approach provided a quick identification of binding sites for further exploration and functional validation. Targeted docking at specific sites (predicted druggable and potentially functional) allows for a more comprehensive analysis of the chemical–biological space. This analysis then guides the selection of drug-like compounds for subsequent experimental validation and development. Thus, the sites identified by blind docking that matched druggable pocket predictions were further analysed by targeted docking using the Chemdiverset library (50,000 compounds). Results were then sorted by binding energy (∆G) scoring, and the top 500 compounds were selected for further analyses.

**Figure 4:**
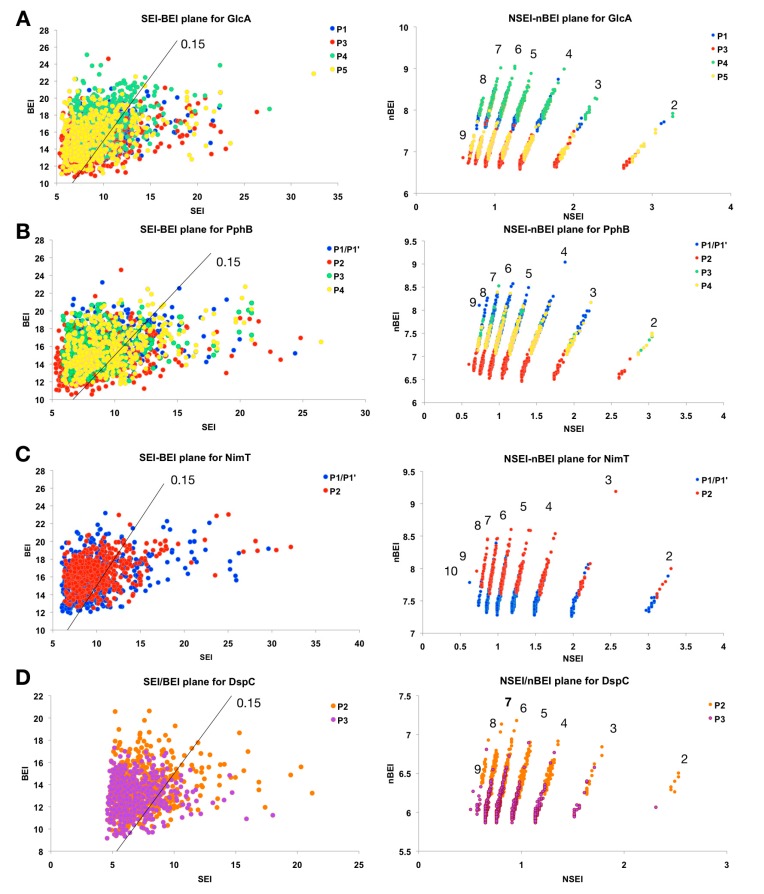
Ligand efficiency plots for binders at the different druggable pockets in AfPPases. On the left, the ligand efficiency plots for clusters obtained from targeted docking. The top 500 compounds (ranked by ∆G) from each cluster are shown. Dots are coloured according to pocket binding for each cluster, as indicated in the plot legend. Line indicates the polar surface area (PSA)/molecular weight (MW) ratio of 0.15 (values defined from the mean PSA and MW of marketed drugs, ([23]). To the right of the line, compounds occupy a favourable chemical space, were binding efficiency index BEI and the surface-binding efficiency index SEI are maximised close to the diagonal of the SEI/BEI plane. On the right column, the NSEI–nBEI plots are shown. Selected ligands (top 500 ranked by ∆G) are displayed; dots are coloured according to the pocket they bind to as indicated in the plot legend. Each diagonal line of ligands represents a specific NPOL (number of N and O atoms in the compound) as indicated by the number above each line. Plots are shown for GlcA (**A**), PphB (**B**), NimT (**C**), and DspC (**D**).

In addition to the ∆G and the estimated potency (K_i_), VSpipe provides the user with a list of several physicochemical parameters and the corresponding ligand efficiency index (LEI) plots. These plots correlate the efficiency of the ligands to their physicochemical properties [22]. For example, the SEI–BEI ligand efficiency plot (Figure 4) correlates the estimated K_i_ of the ligands to their polar surface area (PSA) and molecular weight (MW), where SEI is the surface-binding efficiency index respect to PSA and BEI is the binding efficiency index related to the MW. The most drug-like compounds (those with higher probability of having favourable pharmacokinetic properties and oral bioavailability) will be placed at the upper right quadrant of the plot, with high SEI and BEI values [22,23,24].

For example, GlcA ligands obtained from the targeted docking at P1–P5 were analysed from the SEI/BEI plots (Figure 4A). Overall, ligands show a wide distribution along both axes, with the majority of them distributed around SEI values between 5 and 15 and BEI between 12 and 22. The distribution of P4 ligands (C-terminal groove binders) is shifted towards a higher BEI, suggesting higher binding efficiency. On the other hand, P3 ligands occupy the least favourable chemical drug space, with most compounds located at the left bottom of the plot near the origin, indicating relatively low binding affinities or high polar surface area and molecular weight.

A better discrimination between clusters is observed in the NSEI/nBEI plot, where compounds are sorted by the number of polar atoms, nitrogen, and oxygen (NPOL). Each of these NPOL planes contains compounds with the same number of polar atoms (2, 3, 4, 5, 6, etc.). Polarity increases anti-clockwise, whilst ligand efficiency increases along each NPOL from bottom to top [23,25]. For GlcA, most ligands distribute along NPOL 4 or higher, a trend also observed for the other AfPPases (Figure 4), reflecting the surface polarity of these proteins. The distribution of P4 binders for GlcA is clearly shifted upwards, indicating higher efficiency ligands than those compounds in the other three clusters for the same NPOL. In contrast, P3 binders show the lowest efficiency at all NPOL values (Figure 4A).

For PphB, the distribution is very similar for all ligands, with high SEI/BEI scorers found binding to all pockets, including the active site (P1/P1′), although P2 binders occupy a lower efficiency region, as apparent in the NSEI–nBEI plot. This suggests that P2 is the least druggable site in PphB. In contrast to GlcA, the active site (P1/P1′) in PphB appears more druggable than the rest of sites (Figure 4B).

In the case of NimT and DspC, the distributions in the SEI/BEI plots are narrower and skewed towards the origin of the SEI axis, indicating that binders are more polar. The NSEI–nBEI plot for NimT shows that P2 binders (protein interaction site) clearly occupy a more drug-like chemical space compared to that of the P1/P1′ binders (the extended active site) (Figure 4C). Unlike the STPs, it is more difficult to draw a direct comparison between the two DUSPs, mainly due to active site binders not being identified in the DspC model. However, for DspC binders, LEI values are in general lower than for the rest of AfPPases and occupy a less favourable chemical space (low efficiency) (Figure 4D). A summary of the top scoring compounds for each cluster is found in Supplementary Table S3.

Our analyses indicate that, for the active site, binders have a less favourable LEI distribution than that of binders to other druggable pockets. Thus, the alternative druggable pockets offer better opportunities for the development of drug-like molecules (i.e., P4 for GlcA, P2 for NimT). In contrast, for PphB, the active site appears to be a good druggable option.

### 2.7. Protein–Protein Interaction Sites and Matching with VS Clusters

Currently, there is no information on the importance of the different AfPPase druggable pockets identified in our analyses. However, some hypotheses may be formulated on the basis of their homology to the well-characterised human homologues, for which their function and biological partners have been extensively studied. Thus, it is safe to assume that the active site pocket will be functionally important, as the key catalytic residues are conserved in the AfPPases. In addition, there is a significant amount of information on protein–protein interaction regions from the structures of human homologues in complex with biological partners and inhibitors that define critical binding interfaces. A number of binding regions have been described for hPP1, which are conserved in the *Saccharomyces cerevisiae* PP1-like phosphatase Ppz [26]. These are the: RVxF, ϕϕ, Arg, SILK, NIPP1 (Nuclear inhibitor of protein phosphatase 1)-helix, MYPT1(myosin phosphatase target subunit 1)-helix, and the I-2 (inhibitor 2)-helix binding sites. All of them are also present in GlcA (conserved > 90% except for the NIPP1 and MYPT1 binding sites which are conserved 86% and 75%, see Table 3).

**Figure 5:**
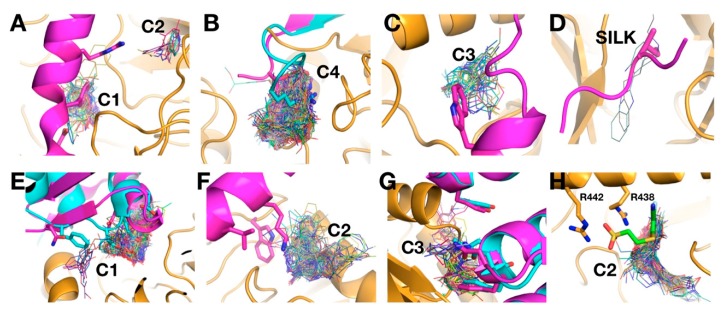
VSpipe blind docking ligand clusters overlap with protein–protein interaction sites. Blind docking clusters in GlcA, PphB, and NimT are shown on molecular models of the AfPPases (ribbon representation in orange and ligands shown as lines). The clusters match the protein interaction sites observed in the crystal structures of complexes of biological partners of the human homologues. Key binding residues are shown in sticks to emphasise the overlap with the ligand clusters identified by VSpipe. The GlcA model (orange) is superimposed to hPP1 interacting partners: (**A**) PPP1R2 (PDB: 2O8A, magenta) binds to the active site and overlaps with C1, while residues at the upper part of the helix are close to C2 in the acidic groove; (**B**) PPP1R15B (PDB: 4V0U, cyan) and PP1G (PDB: 6DNO, magenta) bind at the C-terminal groove and overlap with C4; (**C**) MYPT1, myosin-binding subunit (Protein phosphatase 1 regulatory subunit 12A) (PDB: 1S70) binds at the side pocket and a key tryptophan residue matches C3; (**D**) binding of PP1G at the SILK motif binding site matches with a low density cluster. The PphB model (orange) is superimposed to hPP2A interacting partners: (**E**) the PPME1, Protein phosphatase 2a specific methylesterase, (PDB: 3C5W, magenta) and LCMT-1, Leucine carboxyl methyltransferase, (PDB: 3P71, cyan) binding helices overlap with C1 at the active site; (**F**) SGOL1, Shugoshin-like 1, (PDB: 3FGA) binds to the side pocket and overlaps with C2; (**G**) PP2R1, Protein Phosphatase 2 regulatory subunit, (PDB: 5W0W, magenta) and PP2R1, Protein Phosphatase 2 regulatory subunit, (PDB: 2IE3, cyan) binds to the back of the protein (near P3) overlapping with C3. (**H**) NimT (orange) superimposed to the hCDC25B inhibitor (PDB: 4WH9, sticks in green and atom-type colouring) bound to the hCDK2 (human cyclin dependent kinase 2) kinase interaction site. The position of the inhibitor overlaps with C2. The side chains of the conserved R442 and R438 that participate in the binding are shown as sticks.

Interestingly, most of these protein binding regions map to druggable pockets in GlcA, P1: Active site, and I2-helix binding site, P3: NIPP1-helix and MYPT1-helix, P4: C-terminal groove and Arg-binding site, and P5: SILK binding site (Figure 5). P1 and P4 are fully conserved between hPP1 and GlcA (Table 3), but several residue substitutions affect P2 (Q198 in hPP1 is C197 in GlcA, Q181 to N180 and I189 to V188, E230 to D229, A233 to S232, K234 to R233, H237 to Q236, and L241 to M240), suggesting that specific interactions may somehow be different, although this will only become evident once the structure of GlcA is available.

Importantly, the VS clusters identified overlap with the binding interfaces reported from the crystal structures (Figure 5). For example, the PPP1R2 binding site overlaps with C1 and C2 clusters in GlcA. PPP1R15B and PP1G overlap with C4, PPP1R12 (MYPT1) binding sites overlaps with C3 and PP1G binds at the SILK binding site where it overlaps with a low-density cluster (Figure 5A–D). Similar matching of binding interfaces in hPP2A with VS clusters in PphB are observed: LMCT1 (Leucine carboxyl methyltransferase) and PPME1 (Protein phosphatase 2a specific methylesterase) bind to the active site overlapping the C1 cluster; SGOL1 (Shugoshin-like 1) binding site overlaps with C2 and PP2R1 (Protein Phosphatase 2 regulatory subunit 1) binding sites overlap with C3 (Figure 5E–G).

For the NimT orthologue, hCDC25B, the interacting interface with CDK2 (Cyclin dependent kinase 2) [27,28] has been described, and compounds that bind in this region disrupt dephosphorylation of CDK2 in vitro ([21]). This interface contains two critical arginine residues (R488 and R492) [27,28] essential for interactions with the D206 residue of CDK2 [28]). These arginine residues are conserved in NimT (R438 and R442), as well as the aspartic acid in the *A. fumigatus* orthologue of CDK2 (AfuA_6G07980). The hCDC25B inhibitor-binding site [21] overlaps with the C2 cluster at P2 (Figure 5H). However, P2 in NimT is larger and overall conservation with the analogous pocket in hCDC25B is relatively low (30%), indicating that development of specific NimT inhibitors at P2 may be feasible.

Thus, the VS clusters revealed binding sites that may be relevant for functional interactions in the AfPPases. Supporting this hypothesis is the conservation of many reported human PPase substrates and regulatory subunits in *A. fumigatus* (Supplementary Tables S4 and S5). These are also present in *S. cerevisiae*, where interactions have been experimentally validated. For example, the orthologue of regulatory subunit PPP1R3 (Gac1) interacts with Glc7, the orthologue of GlcA [29]. PP2A interacting proteins are also conserved in *A. fumigatus* (Supplementary Table S5). Assessment of the biological importance of these regions in the AfPPases will validate their suitability as targets for drug development.

The match of these putative biological binding sites with druggable pockets suggests that they could be exploited for the development of protein–protein interaction inhibitors. This offers alternatives to the active site for drug development, particularly since molecules that bind there fall into a more drug-like chemical space, providing promising starting points for further drug development.

## 3. Materials and Methods

### 3.1. Protein Phosphatase Classification

The protein data sets were obtained from the *Aspergillus* genome database (http://www.aspgd.org) and the *Candida* genome database (http://www.candidagenome.org). *S. cerevisiae* sequences were obtained from the *Saccharomyces* genome database (http://www.yeastgenome.org), and the human sequences from the Uniprot database (http://www.uniprot.org). The phosphatase classification was performed using the improved phosphatase ontology tool [16] in a similar way to the method previously used to classify phosphatases from the human and *A. fumigatus* genomes [14] and to generate the TriTryp phosphatome [16]. Each protein identified was manually inspected and validated with literature search and BlastP analysis [18], and later compared to those for the human [30,31] and *S. cerevisiae* reported phosphatases [3,32,33].

### 3.2. Generation of Protein Phosphatase Null Mutants

The protein phosphatase null mutants were generated in the *A. fumigatus* strain MFIG001 (previously known as A1160 Δku80 pyrG+) [34]. Gene replacement cassettes, employing the hygromycin selectable marker, hph, were made and transformation of *A. fumigatus* carried out as described in [35]. The oligonucleotide primers used to generate the replacement cassettes are given in Supplementary Table S2. Integration of the deletion cassette at the correct locus was performed by amplifying from within the gene knockout cassette to flanking regions within the genome but outside the cassette on both flanks using primer combinations (X)P1 and HPHR, and HPHF and (X)P4 where “X” denotes a gene specific designation (Figure 1). To ensure that the strains identified were homokaryotic, a ca. 200 bp region of the coding sequence of the gene of interest was amplified by PCR using primers (X)PPF and (X)PPR. Phenotypic analysis was performed by spot inoculating ca. 1 × 10^3^ spores (in 1 µL) on *Aspergillus* Minimal Media Agar (AMM) and incubating for 48 h at 37 °C.

### 3.3. Molecular Homology Models

The full-length sequences for the *A. fumigatus* phosphatases (AfPPases) were extracted from FungiDB [36] (https://fungidb.org/fungidb). The catalytic domains were first identified using ScanProsite (https://prosite.expasy.org) [37]. The final boundaries were defined using information on secondary structure predictions with JPred [38], (http://www.compbio.dundee.ac.uk/jpred), XtalPred [39] (http://xtalpred.godziklab.org/XtalPred-cgi/xtal.pl) and RONN (https://www.strubi.ox.ac.uk/RONN) [40], and edited to eliminate predicted disordered regions at either the N- or C-terminal end. The final catalytic domain sequences and the 3D structure templates from the closest homologues were then used to create the molecular homology models in Modeller (version 9.20) [20]. All crystal structures used were obtained from the Protein Data Bank (PDB) [19], (https://www.rcsb.org). The basic option in Modeller was used to model FcpA, (from PDB ID: 3EF0, *Schizosaccharomyces pombe* Fcp1), DspC (from PDB ID: 5M43, *C. thermophilum* Yvh1), SsuA (from PDB ID: 3O2S, human Ssu72), and YmrA (from PDB ID: 5GNH, human myotubularin related protein 2 (MTMR2)). The advance option was used for the rest of AfPPases using multiple structure templates, for GlcA (PDB IDs: 4G9J, 5IOH, 1S70, human PP1), PphB (PDB IDs: 2NYL, 3DW8, 2NYM, human PP2A), and NimT (PDB IDs: 1C25, 1QB0, 3OP3, human CDC25B). Modeller produces five models in the modelling development step, the model with the lowest DOPE score is considered the “best structure” and thus chosen for further analysis. DOPE (discrete optimised protein energy) score is a statistical potential that is used to assess the accuracy of homology models. However, the DOPE score is unnormalised with respect to protein size and uses an arbitrary scale; therefore, the score obtained from different proteins cannot be directly compared to each other (e.g., we cannot compare the DOPE score for NimT to the DOPE score from GlcA). The DOPE scores for each model used are found in Table 1.

### 3.4. Virtual Screening with VSpipe

VSpipe [15] is a semi-automated pipeline that uses MGLTools, AutoDock tools [41], OpenBabel [42], and in-house Python and R scripts to perform structure-based virtual screenings. Initially, we used the blind docking option with the Maybridge Ro3 500 fragment library to identify putative ligand binding sites. This is a 500-fragment library, in which compounds are “rule-of-three” (Ro3) compliant. This library is chemically clean (undesirable reactive functionalities removed), highly diverse, and pharmacophore rich. This library is designed to probe sub-pockets of target binding sites. Further details can be found at www.maybridge.com.

Subsequent targeted docking at selected binding sites was done with the Chembridge Chemdiverset library (50,000 compounds), which is a library containing a diverse multisource collection of small molecules with lead-like properties (details in www.cambridgemedchemconsulting.com/DDResources/Hit_iden/frag_collection.html).

We analysed the resulting binding clusters using the ligand efficiency (LEI) plots output by VSpipe: SEI (p(Ki))/(PSA/100 Å^2^) vs. BEI (p(Ki))/MW (kDa), and NSEI (−log_10_(Ki/NPOL)) vs. nBEI (−log_10_(Ki/NHEA)). These plots help to visually define the chemical–biological space for each cluster of ligands obtained from the targeted docking. Blind docking was performed with AutoDock Vina as implemented in VSpipe with a box size that covers the whole protein and a grid spacing of 0.375 Å. For targeted docking, VSpipe lets the user decide whether either AutoDock Vina [43] or AutoDock 4.2 [41] are to be used for the docking step of the VS. In this study, we chose AutoDock 4.2. We then calculated the grid boxes, so they were centred around each pocket, although it is noteworthy that box size varied depending on the pocket chosen. All computational tasks were carried out at the Computational Shared Facility (CSF) at the University of Manchester.

### 3.5. Pocket Predictions

Pocket druggability predictions were performed using the PockDrug-Server [17]. The software predicts protein pockets using a number of descriptors that combine geometric and physicochemical criteria to provide a mean druggability score. Pockets with druggability scores of 0.5 or higher were considered as druggable pockets and were then inspected graphically in PyMol. Those pockets located in the interior of the protein or that were not surface exposed were removed from subsequent analyses. Small pockets with < 10 residues (decoys) were also eliminated from the analysis.

All protein structure images were created with MacPyMOL: PyMOL v1.8.0.3 Enhanced for Mac OS X (Schrödinger LLC, NY, USA).

### 3.6. Sample Availability

The Linux version of VSpipe-local mode and documentation are available at https://github.com/sabifo4/VSpipe. The VSpipe-cluster mode is available upon request.

## 4. Conclusions

Functional information on *A. fumigatus* phosphatases is limited [3,10,13]. Although some functional roles can be inferred by the studies done in *Candida* spp. or *S. cerevisiae* orthologues, the true biological roles remain largely unknown. In this study, we have identified three new phosphatase genes (TepA, YmrA, and PPEFA) and confirmed that seven AfPPases are important for viability.

VS with VSpipe [15] on the essential AfPPases, identified potential functional sites in these AfPPases for further experimental evaluation. Putative functional sites were inferred from the matches of VS ligand clusters with protein–protein interaction interfaces described for the human homologues and supported by the conservation of the interacting partners in *A. fumigatus* and in *S. cerevisiae*, where interactions have been experimentally demonstrated.

Further targeted docking analyses with VSpipe, at defined druggable sites, suggested that different modes of inhibition (active site and protein–protein interaction) could be exploited to target the AfPPases, and identified initial hits for drug development in a favourable chemical space.

## Figures and Tables

**Table 1:** *Aspergillus fumigatus* phosphatases (AfPPases) analysed in this study. The human homologues are indicated. Protein Data Bank (PDB) IDs are template structures used for homology model building. Identity and coverage for both the full length and the sequences used in the models were calculated from the templates sequences as marked. H: Human, Ct: *Chaetomium thermophilum*, Sp: *Schizosaccharomyces pombe*. Domain boundaries are the ones used for the homology model building. * dual-specificity phosphatase Yvh1 is the Sp homologue of the dual-specificity phosphatase DspC, Dual-specificity phosphatase DUSP12 is the human homologue. The model scores correspond to the Modeller objective function score (discrete optimised protein energy (DOPE) score) provided by Modeller [20] for the best model. PP2AC: catalytic domain of protein phosphatase 2A, PP1CB: catalytic domain of protein phosphatase 1 β, Fcp1: TFIIF-stimulated CTD phosphatase 1, CDC25B: cell division control protein 25B, Yvh1: dual-specificity phosphatase, DUSP12: Dual-specificity phosphatase 12, Ssu72: RAN polymerase II C-terminal domain phosphatase SSU72, MTMR2: Myotubularin-related protein 2.

Af PPase	Human Homologue	Full-Length Identity/Coverage (%)	Model Identity/Coverage (%)	Template (PDB ID)	Model Boundaries	Model Scores
PphB	PP2AC	84/94 (H)	86/96	2NYL, 3DW8, 2NYM	S19-P311	−37,801.93
GlcA	PP1CB	84/100 (H)	86/100	4G9J, 5IOH, 1S70	M1-E299	−39,831.18
FcpA	Fcp1	44/75 (Sp)	44/99	3EF0	R145-P602	−34,085.44
NimT	CDC25B	43/44 (H)	43/87	1C25, 1QB0, 3OP3	D333-K504	−18,191.54
DspC	* Yvh1/DUSP12	40/95 (Ct)	39/97	5M43	M1-H153	−17,151.59
SsuA	Ssu72	45/75 (H)	45/87	3O2S	S48-L287	−22,385.70
YmrA	MTMR2	48/77 (H)	34/89	5GNH	121-647	−65,336.31

**Table 2:** PockDrug predicted pockets in the AfPPases. Values are druggability scores generated by PockDrug-Server [17]. In bold, the pockets that match ligand clusters from VSpipe blind docking. Pockets used in the targeted docking are marked with “*”. Underlined are pockets involved in protein–protein interactions. P1 is the active site and P1′ is a secondary pocket connected to the active site P1.

PPase	P1/P1′	P 2	P3	P 4	P5
**NimT**	0.55 */0.68 *	0.60 *			
**DspC**		0.55 *	0.69 *		
**PphB**	0.64 */0.6 *	0.73 *	0.63 *	0.70 *	
**GlcA**	0.57 *		0.64 *	0.63 *	0.71 *

**Table 3:** Pocket conservation between AfPPases and human homologues. Conservation of pockets was calculated using the residues defined by PockDrug and comparing these residues to their human counterparts.

AfPPase	Conservation (%)				
	P1/P1′	P2	P3	P4	P5
**NimT**	38	30			
**DspC**		36	31		
**GlcA**	100		96	100	82
**PphB**	100	100	66	90	

## Supplementary Materials

Supplementary materials can be found at https://www.mdpi.com/1422-0067/20/18/4636/s1.
