# Supplementary material for: Identification of Functional and Druggable Sites in Aspergillus fumigatus Essential Phosphatases by Virtual Screening
Source: Int J Mol Sci. 2019 Sep 19;20(18):4636. doi: 10.3390/ijms20184636 (PMC6769645; doi:10.3390/ijms20184636)
Supplement: Supplementary file 1 [file ijms-20-04636-s001.pdf]

Supplementary Material

Supplementary Table S1. Fungi phosphatases studied in this work. *A. fumigatus, C. albicans* phosphatases were identified by the ontology classification tool and then confirmed by Blastp into the FungiDB and CGD. *C. neoformans* were obtained from Blastp searching. STP is serine/threonine phosphatase; PTP is phospho-tyrosine phosphatase; LP is lipid phosphatase.

A. fumigatus	Phosphatase Family	Gene Name	Viable in AF	*C. albicans*	Gene Name	Viable in CA*	*C. neoformans*
AFUA_1g13040	PTP	**dspA**	Y	None			CNAG_00263
AFUA_1g05640	PTP/LP	**ymrA**	N	C1_08860C_A	uncharacterised	Y	CNAG_00390
AFUA_1g03540	PTP	**dspB**	Y	C1_04640W_A	uncharacterised	Y	CNAG_00498
AFUA_3g12250	PTP	**cdcA**	Y	C6_00670W_A	CDC14		CNAG_00498
AFUA_2g03760	PTP	**ssuA**	N	CR_03210C_A	SSU72	N	CNAG_01054
AFUA_4g07080	PTP	**dspC**	N	CR_03570C_A	YVH1	N	CNAG_01203
AFUA_2g02760	PTP	**dspD**	Y	C1_10000C_A	CPP1	Y	CNAG_01357
AFUA_6g08200	PTP	**nimT**	N	C3_00800W_A	MIH1	Y	CNAG_01572
AFUA_4g07000	PTP	**yphA**	Y	CR_06830C_A	SIW14	Y	CNAG_03296
AFUA_6g06650	PTP/LP	**ptyA**	Y	C2_09250W_A	uncharacterised	Y	CNAG_03296
AFUA_2g01880	PTP	**ltpA**	Y	C1_08260C_A	LTP1	Y	CNAG_03871
AFUA_2g11990	PTP/LP	**tepA**	Y	C7_02650W_A	TEP1	Y	CNAG_04833
AFUA_3g10970	PTP	**ptpB**	Y	CR_08070W_A	PTP1	Y	CNAG_05155
AFUA_4g04710	PTP	**ptpA**	Y	CR_10340W_A	PTP3	Y	CNAG_05155
AFUA_5g11690	PTP	**pps1**	Y	C7_00840C_A	PPS1	Y	CNAG_05639
AFUA_5g12010	STP	**pphA**	Y	CR_03800C_A	PPH3	Y	CNAG_00408
AFUA_1g06860	STP	**ptcF**	Y	CR_08160W_A	PTC5	Y	CNAG_00427
AFUA_4g00720	STP	**ptcH**	Y	CR_07770C_A	PTC6	Y	CNAG_00500
AFUA_5g13340	STP	**ptcG**	Y	C1_09260C_A	PTC1	Y	CNAG_00500
AFUA_1g09460	STP	**nemA**	Y	C4_01300W_A	NEM1	Y	CNAG_01177
AFUA_6g11470	STP	**sitA**	Y	C1_04380W_A	SIT4	Y	CNAG_01436
AFUA_5g06700	STP	**pptA**	Y	C3_01710C_A	PPT1	Y	CNAG_01496
AFUA_6g10830	STP	**pphB**	N	C3_01600W_A	PPH21		CNAG_02177
AFUA_5g11370	STP	**ppgA**	Y	C3_01600W_A	PPH21		CNAG_02236
AFUA_5g08620	STP	**ppefA**	Y	C7_03500W_A	uncharacterised	Y	CNAG_02470
AFUA_1g09280	STP	**ptcB**	Y	CR_01520W_A	PTC2	y	CNAG_03052
AFUA_2g03890	STP	**ptcE**	Y	CR_01520W_A	PTC2	Y	CNAG_03052
AFUA_8g04580	STP	**ppmA**	Y	C4_00340W_A	PTC7	Y	CNAG_03541
AFUA_2g03950	STP	**phzA**	Y	CR_06420W_A	PPZ1	Y	CNAG_03673
AFUA_1g04950	STP	**glcA**	N	CR_07650W_A	GLC7	N	CNAG_03706
AFUA_3g11410	STP	**fcpA**	N	C3_07460W_A	FCP1	Y	CNAG_04120
AFUA_1g04790	STP	**psrA**	Y	C3_00570C_A	PSR1	Y	CNAG_04224
AFUA_5g09360	STP	**calA/ cnaA**	Y	C1_00730C_A	CMP1	Y	CNAG_04796
AFUA_1g15800	STP	**ptcA**	Y	CR_07770C_A	PTC6	Y	CNAG_06418
AFUA_5g13740	STP	**ptcD**	Y	None			None

* Based on large scale null data provided at CGD

Supplementary Table S2. Oligonucleotides used in this study.

**Oligo Name**	**Oligo Sequence**
(AFUA_1g05640)P1	GGGTAACCACACTACTGCCG
(AFUA_1g05640)P2	TAGTTCTGTTACCGAGCCGGCAAACCTTAGCGATCCGTGT
(AFUA_1g05640)P3	GCTCTGAACGATATGCTCCAACTGCGACGATTACAACAAAGG
(AFUA_1g05640)P4	CTTGAGCAGATTCCCCAAAG
(AFUA_1g05640)P5	TTAATCCCTACCGAACGACG
(AFUA_1g05640)P6	CCGCTCTCCATGATGAATCT
(AFUA_2g11990)P1	GGGTTCAACACTACGTGCAA
(AFUA_2g11990)P2	TAGTTCTGTTACCGAGCCGGGATGGGAGAGGAAGAAAGGG
(AFUA_2g11990)P3	GCTCTGAACGATATGCTCCAACACCAAGCTTGTTCGTCGTTT
(AFUA_2g11990)P4	GAGTATCTCCGGCTCATGGA
(AFUA_2g11990)P5	CATCTTCCATGCGGAACTTT
(AFUA_2g11990)P6	GCTTTCTGTCGTGGATGGAT
(AFUA_5g08620)P1	GTACCGATGACCCTCTGTCG
(AFUA_5g08620)P2	TAGTTCTGTTACCGAGCCGGGCAAGGTCGGAGATTTCGTA
(AFUA_5g08620)P3	GCTCTGAACGATATGCTCCAACGGTGTTTGGGCGTCTACAAT
(AFUA_5g08620)P4	TGCTCTCACTTTCCATCACG
(AFUA_5g08620)P5	GCTGTTTTCGGATACACGGT
(AFUA_5g08620)P6	TATTTCTGCGTCAGGCACAC
(AFUA_5g11370)P1	ATGTGACCGAAAGAGCGACT
(AFUA_5g11370)P2	TAGTTCTGTTACCGAGCCGGAATGGACAACGTGCCAAACT
(AFUA_5g11370)P3	GCTCTGAACGATATGCTCCAACAGTACTTCCCGATGCAGAGC
(AFUA_5g11370)P4	GATCTCGTTGCCTCGAAGAC
(AFUA_5g11370)P5	GGCGAAGACTAAAACGCTTG
(AFUA_5g11370)P6	ACCAGAATGACGAATCCCTG
(AFUA_2g03760)P1	CGGTGGGCAGAAATCTTCCT
(AFUA_2g03760)P2	TAGTTCTGTTACCGAGCCGGGGAAGTGACAGAGTGGGAGC
(AFUA_2g03760)P3	GCTCTGAACGATATGCTCCAACAACTCCAACTAAGCAAAAGGGT
(AFUA_2g03760)P4	TTGGCCTGAACATCACCTCG
(AFUA_2g03760)P5	TCATGAGTTAGGCGTGACGG
(AFUA_2g03760)P6	ACTGGAACCCTTCGCATGAC
(AFUA_6g08200)P1	ATGGACTATGGAGTCGTCCG
(AFUA_6g08200)P2	TAGTTCTGTTACCGAGCCGGGGGATTTCCTCGGTAAAGGA
(AFUA_6g08200)P3	GCTCTGAACGATATGCTCCAACAGCCGGTTGACTTTCCTTTT
(AFUA_6g08200)P4	CGATGCCTCAGGTTTTCTTC
(AFUA_6g08200)P5	GCGCCCTACCTACGTAACAA
(AFUA_6g08200)P6	CCCAATTTTCTCGCCAAATA
AFUA_1G05640_PPF	GAGCAGCGAAAAGTGGTTTC
AFUA_1G05640_PPR	GGGGACTGTCGGAGTCATAA
AFUA_2G11990_PPF	GAACTTCCTCGACACCAAGC
AFUA_2G11990_PPR	CCATCTGGACCATCGAGTCT
AFUA_5G08620_PPF	ATTCACTGCCGAGATTCGAT
AFUA_5G08620_PPR	CACTGTCCGCATCAATCAGT
AFUA_5G11370_PPF	TGAAGGAGAGCAATGTGGTG
AFUA_5G11370_PPR	CGTAGTCGCCTACACAACGA

```
1S70        AGHMADGELNVDSLITRLLEVRGCRPGKIVQMTEAEVRGLCIKSREIFLSQPILLELEAP  60
4G9J        -GMSDSEKLNLDSIIGRLLEVQGSRPGKNVQLTENEIRGLCLKSREIFLSQPILLELEAP  59
5IOH        ---GHMGSLNLDSIIGRLLEVQGSRPGKNVQLTENEIRGLCLKSREIFLSQPILLELEAP  57
GlcA        ---MADQEVDLDNIIDRLLEVRGSRPGKQVQLLEAEIRYLCTKAREIFISQPILLELEAP  57
                   .:::*.:* *****:*.**** **: * *:* ** *:****:***********

1S70        LKICGDIHGQYTDLLRLFEYGGFPPEANYLFLGDYVDRGKQSLETICLLLAYKIKYPENF  120
4G9J        LKICGDIHGQYYDLLRLFEYGGFPPESNYLFLGDYVDRGKQSLETICLLLAYKIKYPENF  119
5IOH        LKICGDIHGQYYDLLRLFEYGGFPPESNYLFLGDYVDRGKQSLETICLLLAYKIKYPENF  117
GlcA        IKICGDIHGQYYDLLRLFEYGGFPPEANYLFLGDYVDRGKQSLETICLLLAYKIKYPENF  117
            :********** **************:*********************************

1S70        FLLRGNHECASINRIYGFYDECKRRFNIKLWKTFTDCFNCLPIAAIVDEKIFCCHGGLSP  180
4G9J        FLLRGNHECASINRIYGFYDECKRRYNIKLWKTFTDCFNCLPIAAIVDEKIFCCHGGLSP  179
5IOH        FLLRGNHECASINRIYGFYDECKRRYNIKLWKTFTDCFNCLPIAAIVDEKIFCCHGGLSP  177
GlcA        FVLRGNHECASINRIYGFYDECKRRYNIKLWKTFTDCFNCLPIAAIIDEKIFTMHGGLSP  177
            *:***********************:********************:*****  ******

1S70        DLQSMEQIRRIMRPTDVPDTGLLCDLLWSDPDKDVQGWGENDRGVSFTFGADVVSKFLNR  240
4G9J        DLQSMEQIRRIMRPTDVPDQGLLCDLLWSDPDKDVQGWGENDRGVSFTFGAEVVAKFLHK  239
5IOH        DLQSMEQIRRIMRPTDVPDQGLLCDLLWSDPDKDVQGWGENDRGVSFTFGAEVVAKFLHK  237
GlcA        DLNSMEQIRRVMRPTDIPDCGLLCDLLWSDPDKDITGWSENDRGVSFTFGPDVVSRFLQK  237
            **:*******:*****:** **************: **.*********** :**::**::

1S70        HDLDLICRAHQVVEDGYEFFAKRQLVTLFSAPNYCGEFDNAGGMMSVDETLMCSFQILKP  300
4G9J        HDLDLICRAHQVVEDGYEFFAKRQLVTLFSAPNYCGEFDNAGAMMSVDETLMCSFQILKP  299
5IOH        HDLDLICRAHQVVEDGYEFFAKRQLVTLFSAPNYCGEFDNAGAMMSVDETLMCSFQILKP  297
GlcA        HDMDLICRAHQVVEDGYEFFSKRQLVTLFSAPNYCGEFDNAGAMMSVDESLLCSFQILKP  297
            **:*****************:*********************.******:*:********

1S70        SEKKAK--YQYGGLN-SGRPVTPPRTANPPKKR     330
4G9J        ADKNKGKYGQFSGLNPGGRPITPPRNSAKAKK-     331
5IOH        AD-------------------------------     299
GlcA        AE-------------------------------     299
            ::
```

(**A**)

```
2NYL        ---DEKVFTKELDQWIEQLNECKQLSESQVKSLCEKAKEILTKESNVQEVRCPVTVCGDV  57
3DW8        --MDEKVFTKELDQWIEQLNECKQLSESQVKSLCEKAKEILTKESNVQEVRCPVTVCGDV  58
2NYM        ---DEKVFTKELDQWIEQLNECKQLSESQVKSLCEKAKEILTKESNVQEVRCPVTVCGDV  57
PphB        SPTTDPGSIPTLDGWIENLMACKQLAEDDVRRLCDRAREVLQEESNVQPVKCPVTVCGDI  60
                :      ** ***:*  ****:*.:*: **::*:*:* :***** *:********:

2NYL        HGQFHDLMELFRIGGKSPDTNYLFMGDYVDRGYYSVETVTLLVALKVRYRERITILRGNH  117
3DW8        HGQFHDLMELFRIGGKSPDTNYLFMGDYVDRGYYSVETVTLLVALKVRYRERITILRGNH  118
2NYM        HGQFHDLMELFRIGGKSPDTNYLFMGDYVDRGYYSVETVTLLVALKVRYRERITILRGNH  117
PphB        HGQFHDLMELFRIGGPNPDTNYLFMGDYVDRGYYSVETVTLLVCLKIRYPQRITILRGNH  120
            *************** .**************************.**:** :*********

2NYL        ESRQITQVYGFYDECLRKYGNANVWKYFTDLFDYLPLTALVDGQIFCLHGGLSPSIDTLD  177
3DW8        ESRQITQVYGFYDECLRKYGNANVWKYFTDLFDYLPLTALVDGQIFCLHGGLSPSIDTLD  178
2NYM        ESRQITQVYGFYDECLRKYGNANVWKYFTDLFDYLPLTALVDGQIFCLHGGLSPSIDTLD  177
PphB        ESRQITQVYGFYDECLRKYGNANVWKYFTDLFDYLPLTALIENQIFCLHGGLSPSIDTLD  180
            ****************************************::.*****************

2NYL        HIRALDRLQEVPHEGPMCDLLWSDPDDRGGWGISPRGAGYTFGQDISETFNHANGLTLVS  237
3DW8        HIRALDRLQEVPHEGPMCDLLWSDPDDRGGWGISPRGAGYTFGQDISETFNHANGLTLVS  238
2NYM        HIRALDRLQEVPHEGPMCDLLWSDPDDRGGWGISPRGAGYTFGQDISETFNHANGLTLVS  237
PphB        NIRSLDRIQEVPHEGPMCDLLWSDPDDRCGWGISPRGAGYTFGQDISEAFNHNNGLTLVA  240
            :**:***:******************** *******************:*** ******:

2NYL        RAHQLVMEGYNWCHDRNVVTIFSAPNYCYRCGNQAAIMELDDTLKYSFLQFDPAPR----  293
3DW8        RAHQLVMEGYNWCHDRNVVTIFSAPNYCYRCGNQAAIMELDDTLKYSFLQFDPAPRRGEP  298
2NYM        RAHQLVMEGYNWCHDRNVVTIFSAPNYCYRCGNQAAIMELDDTLKYSFLQFDPAPR----  293
PphB        RAHQLVMEGYNWSQDRNVVTIFSAPNYCYRCGNQAAIMEIDEHLKYTFLQFDP-------  293
            ************.:*************************:*: ***:******

2NYL        -----------   293
3DW8        HVTRRTPDYFL   309
2NYM        -----------   293
PphB        -----------   293
```

(**B**)

```
NimT        ---------------------------DVDTTPSLQLPHF-------IPEDSADTLPRID  26
3OP3        MHHHHHHSSGVDLGTENLYFQSMTQMLEEDSNQGHLIGDFSKVCALPTVSGKHQDLKYVN  60
1C25        ----------------------------------MLIGDFSKGYLFHTVAGKHQDLKYIS  26
1QB0        -----------VLRSKSLCHDEIENL--LDSDHRELIGDYSKAFLLQTVDGKHQDLKYIS  47
                                                : .:          .. : *  :.

NimT        KTILVDLMDGKYNDRFDHIMVVDCRFEYEYEGGHINGAVNYNDKEYLAAQLFADP-----  81
3OP3        PETVAALLSGKFQGLIEKFYVIDCRYPYEYLGGHIQGALNLYSQEELFNFFLKKPIVPLD  120
1C25        PEIMASVLNGKFANLIKEFVIIDCRYPYEYEGGHIKGAVNLHMEEEVEDFLLKKPIVPTD  86
1QB0        PETMVALLTGKFSNIVDKFVIVDCRYPYEYEGGHIKTAVNLPLERDAESFLLKSPIAPCS  107
               :. :: **: . ...: ::***: *** ****: *:*   :.     :: .*

NimT        -KPRTALVLHCEYSAHRAPIMAKYIRHRDRAFNVDHYPHLTYPDMYILDGGYSAFFAEHR  140
3OP3        TQKRIIIVFHCEFSSERGPRMCRCLREEDRSL--NQYPALYYPELYILKGGYRDFFPEYM  178
1C25        -GKRVIVVFHCEFSSERGPRMCRYVRERDRLG--NEYPKLHYPELYVLKGGYKEFFMKCQ  143
1QB0        LDKRVILIFHCEFSSERGPRMCRFIRERDRAV--NDYPSLYYPEMYILKGGYKEFFPQHP  165
               *  :::***:*:.*.* *.: :*..**    :.** * **::*:*.***  ** :

NimT        SLCFPQNYVEMNAKEHEFACERGLGKVKQRSK--------------        172
3OP3        ELCEPQSYCPMHHQDHKTELLRCRSQSKVQEGERQLRE--------        216
1C25        SYCEPPSYRPMHHEDFKE----------------------------        161
1QB0        NFCEPQDYRPMNHEAFKDELKTFRLKTRSWAGERSRRELCSRLQDQ        211
            . * * .*  *: : .:
```

(**C**)

```
5M43        MALNRIKGDDELFVGGVFGANRARLIKEHRITHILSVIDHTVDRENEAFRHVKHLSIDID  60
DspC        MAMNKIPGH-DIYIGGILAVKNKAALSRANITHIVSVLR--LNQPDEIFANFQHHCIHAD  57
            **:*:* *. ::::**::..:.   :.. .****:**:   ::: :* * :.:* .*. *

5M43        DMEDQDILIHLPKIVRFIDSGLRGIDPSDSSAVASPGVVLVHCAMGKSRSVTAIIAYLLW  120
DspC        DVGDENLLEHFPSAIKFIQSGLD-----------AGGSVLVHCAMGKSRSATICIAYLLH  106
            *: *:::* *:*. ::**:***            : * ************.*  *****

5M43        KYPYRFGKSDPNISAKEAVSRALEWVRETRPIAGPNDGFMRQLEMWWDMGCPADSDDAVE  180
DspC        QQPSALTP-----------QSALALLRKGRPLCEPNDGFMEQLALYHEMGCPDDVTGH--  153
            : *  :             . **  :*: **:. ******.** :: :**** *  .

5M43        REPAYQRWLYQREVEDAARIGRAPDRLRFEDEAATAEGSHHHHHH 225
DspC        --------------------------------------------- 153
```

(**D**)

Supplementary Figure S1. Omega alignments between *AfPPases*: GlcA (**A**), PphB (**B**), NimT (**C**), DspC (**D**) and template sequences used to create each model. (*) identical residues, (:) residues with strong similar properties, (.) residues with weak similarity.

Supplementary Table S3. Top scoring compounds obtained in the targeted docking with the Chemdiverset library, for each AfPPase and cluster. The table shows the compound structure, compound library ID, molecular weight (Mw), polar surface area (PSA), Gibbs free energy (ΔG) and ligand efficiency indices calculated by VSpipe (BEI, SEI). Compounds show diversity except for those binding at the active site pocket (C1) of GlcA and PphB due to the high conservation between their active sites.

AfPPases	Cluster	Compound Structure	ID	Mw	PSA	ΔG	BEI	SEI
**NimT**	C1	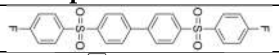	5236413	470.5	68.3	-9.5	14.8	10.2
	C2	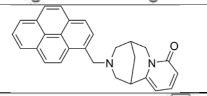	5641450	404.5	25.2	-10.4	19.0	30.5
**PphB**	C1	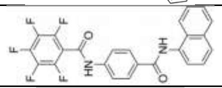	5213442	456.4	58.2	-10.2	16.5	12.9
	C2	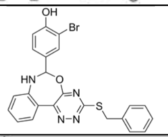	6633679	493.4	80.2	-8.2	12.2	7.5
	C3	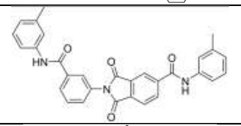	5569062	489.5	95.6	-9.5	14.2	7.3
	C4	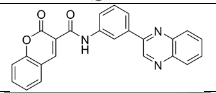	7966036	393.4	85.1	-9.4	17.5	8.1
**GlcA**	C1	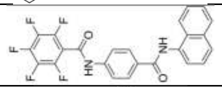	5213442	456.4	58.2	-9.9	15.8	12.4
	C2	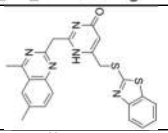	7673412	446.5	96.5	-9.5	15.6	7.2
	C3	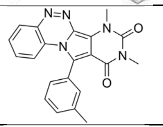	7702995	371.4	74.2	-8.6	16.9	8.5
	C4	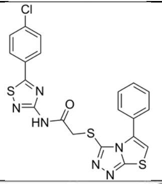	7743093	485.0	85.1	-10.3	15.5	8.8
	C5	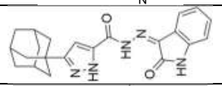	5784178	389.5	99.2	-8.9	16.7	6.6
**DspC**	C2	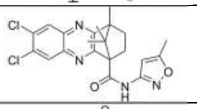	7784277	431.3	80.9	-7.8	13.3	7.1
	C3	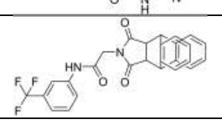	6629705	476.4	66.5	-7.3	11.2	8.0

Supplementary Table S4. Human PP1 biological partners present in the crystal structures and their homologues in *S. cerevisiae, C. albicans* and *A. fumigatus*. Percentage of identity is respect of the human proteins. Validated *S. cerevisiae* PP1 (Glc7) interactions are marked with (*).

PDB	Gene in Human	*S. cerevisiae*	Gene ID	ID/Cover (%)	*C. albicans*	Gene ID	ID/ Cover (%)	*A. fumigatus*	Gene ID	ID/Cover (%)
**1S70**	PPP1R12A (MBS, MYPT1)	Hos4[V]	YIL112W	37/17 %	Uncharacterised protein	CAALFM_C304920CA	30/22 %	Pfs	AFUA_1G01020	23/36 %
**2O8A**	PPP1R2	Glc8*[V]	YMR311C	20/100%	GLC8	CAALFM_C402860WA	23/100 %	Uncharacterised protein	AFUA_5G03700	32/100 %
**3V4Y**	PPP1R8	PML1[V]	YLR016C	14/100 %	Ubiquitin-conjugating protein	CAALFM_C600260WA	50/10 %	SNIP1	AFUA_3G11540	31/100 %
**4MOY**	PPP1R10 (Cat53, Pnuts)	SKI2[V]	YLR398C	33/7 %	SKI2	CAALFM_CR08570WA	6/100 %	Pab1[U]	AFUA_1G04190	41/10 %
**4V0U**	ACTA1	ACT1[U]	YFL039C	88/99 %	ACT1	CAALFM_C113700WA	83/99 %	ACT1	AFUA_6G04740	87/90 %
**6DNO**	PPP1R3A (PP1G)	Gac1*[V]	YOR178C	28/12 %	Gac1	CAALFM_C700660WA	37/8 %	Gac1	AFUA_2G13850	29/19%
**6GHM**	TP53BP2 (ASPP2, BBP)	Nas6[V]	YGR232W	36/11%	Uncharacterised protein	CAALFM_C108700WA	36/10 %	Ankyrin repeat protein	AFUA_8G02140	31/14%

*: Glc7 partners (SGC database); V: KO viable; U: KO unviable

Supplementary Table S5. Human PP2A biological partners present in the crystal structures and their homologues in *S. cerevisiae*, *C. albicans* and *A. fumigatus*. Percentage of identity is respect of the human proteins. Validated *S. cerevisiae* PP2A (Pph21/22) interactions are marked with (*).

**PDB**	**Gene in human**	***S. cerevisiae***	**Gene ID**	**ID/Cover (%)**	***C. albicans***	**Gene ID**	**ID/ Cover (%)**	***A. fumigatus***	**Gene ID**	**ID/Cover (%)**
**2IE3**	PPP2R1A	TPD3[*V]	YAL016W	44/98 %	TPD3	CAALFM_C306910CA	42/97 %	PPP2R1A[U]	AFUA_1G05610	53/98 %
**2NPP**	PPP2R5C	RTS1[*V]	YOR014W	56/78 %	RTS1	CAALFM_C206420CA	57/81 %	SSC1[V]	AFUA_5G02560	67/77 %
**3DW8**	PPP2R2A	CDC55[*V]	YGL190C	56/88 %	CDC55	CAALFM_C201600CA	58/97 %	PPP2RB[V]	AFUA_8G05560	57/96 %
**4IYP**	IGBP1 (IBP1)	Tap42[*U]	YMR028W	26/86 %	-	CAALFM_C401670CA	23/96 %	TapA[U]	AFUA_5G11780	26/90 %
**5W0W**	Tip41-like (TIPRL)	Tip41[*V]	YPR040W	35/89 %	Tip41	CAALFM_C504520WA	39/93 %	TipA[V]	AFUA_2G07540	41/89 %
**3C5W**	PPME1 (PME1)	PPE1/ YmS2[*V]	YHR075C	29/93 %	YmS2	CAALFM_C201520WA	32/92 %	PPE1[V]	AFUA_1G03080	37/80 %
**4I5L**	PPP2R2D (CDC6)	CDC6[U]	YJL194W	27/70 %	CDC6	CAALFM_C112220WA	37/41 %	CDC6	AFUA_7G04310	30/65 %
**4LAC**	PTPA	RRD1[*V]	YIL153W	43/75 %	RRD1	CAALFM_C307080WA	39/91 %	RRD2[V]	AFUA_1G13420	32/93 %

*: Pph21/22 partners (SGC database); V: KO viable; U: KO unviable

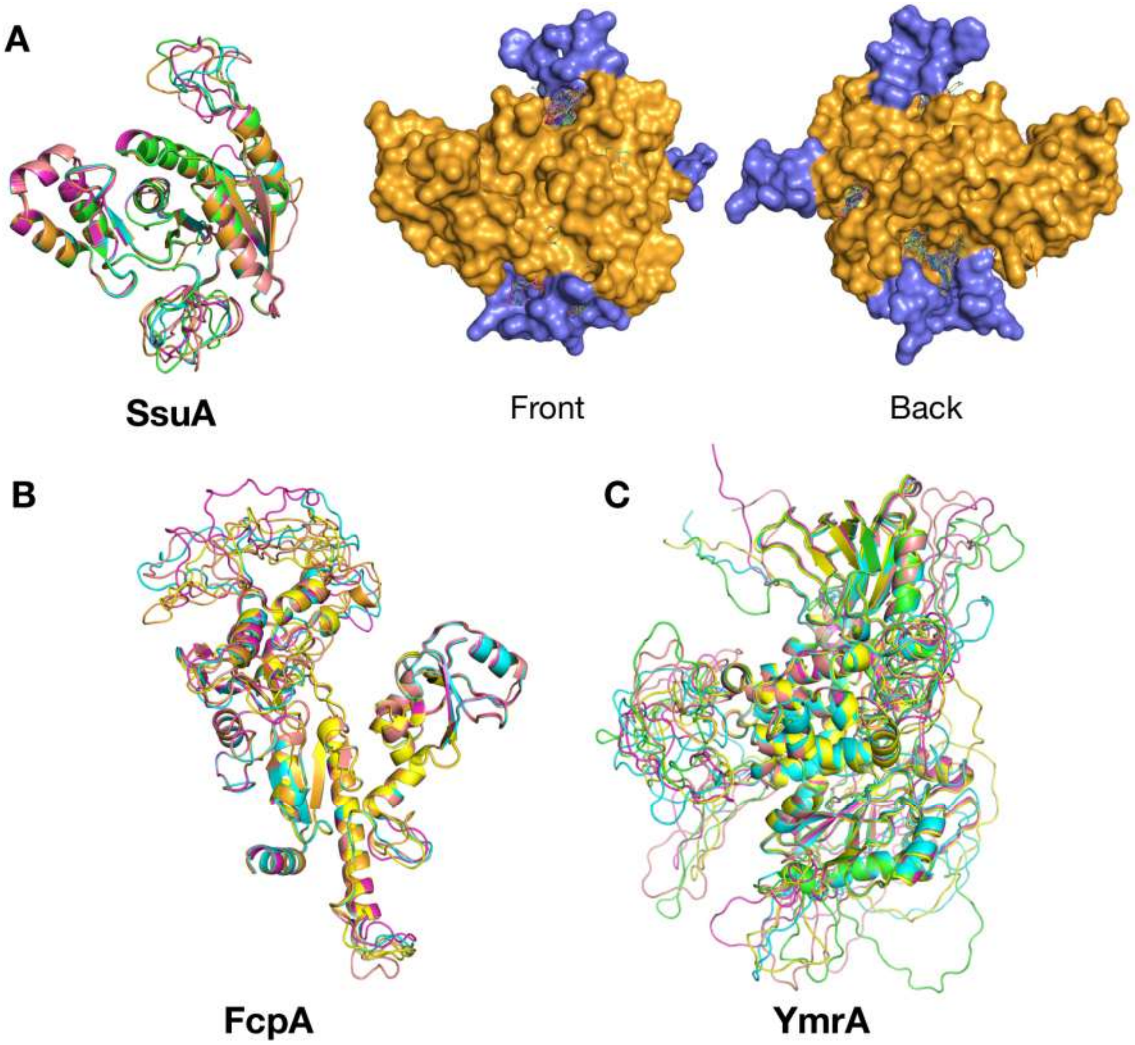

Supplementary Figure S2. Molecular models for AfPPases. (**A**). Cartoon representation of the molecular models generated by Modeller for SsuA superimposed. Insertions regions, respect to the template structure, appear as disordered loops at the top and bottom of the phosphatase core. To the right is the surface representation of SsuA (orange), insertion regions are coloured lilac and blind docking ligand clusters obtained with VSpipe are shown in lines binding near the insertion regions. (**B**). Molecular models for FcpA are shown in cartoon representation, unstructured insertions are visible at the top and bottom of the core of the structure. (**C**). Molecular models for YmrA shown as cartoons and unstructured regions shown in different regions of the protein.
